# Outcomes of children aged 2–59 months with chest indrawing pneumonia managed on an outpatient basis in selected primary health facilities in Zambia

**DOI:** 10.7189/jogh.15.04089

**Published:** 2025-07-11

**Authors:** Choolwe Jacobs, Chipo Nkwemu, Bryan Bakele Ngambi, Vichael Silavwe, Shamim Ahmad Qazi, Yasir Bin Nisar

**Affiliations:** 1 Department of Epidemiology and Biostatistics, School of Public Health, University of Zambia, Lusaka, Zambia; 2 Department of Strategic Information, Centre for Infectious Disease Research in Zambia, Lusaka, Zambia; 3 Save the Children, Lusaka, Zambia; 4 Department of Child Health Unit, Ministry of Health, Lusaka, Zambia; 5 Independent Consultant, Geneva, Switzerland; 6 Department of Maternal, Newborn, Child and Adolescent Health and Ageing, World Health Organization, Geneva, Switzerland

## Abstract

**Background:**

Zambia has a high burden of child pneumonia, with approximately 6000 children under five dying annually from this condition. We aimed to gather evidence about the outcomes two weeks after enrolment for children 2–59 months with chest indrawing pneumonia who were managed in primary health care facilities in Zambia.

**Methods:**

This was a prospective cohort study conducted between October 2022 and April 2024 in eight primary health care facilities from Lusaka, Chibombo, and Chongwe districts. Children aged 2–59 months with cough and/or breathing difficulty and lower chest indrawing were enrolled, treated on an outpatient basis according to the Integrated Management of Childhood Illness (IMCI) protocol, and followed up on day 15 after enrolment.

**Results:**

We enrolled 335 children in the study (median age of 17 months, 56.4% female). Among them, 63% were aged 12–59 months, 23.6% had a height-for-age z-score of<−3.0, and 10.5% had a weight-for-age z-score of<−3.0. By day 15, 314 children had follow-up data, all of whom survived. Of these, 77.1% were cured, 22.9% were reported as being ‘clinically better’, and none failed therapy. Most children were treated with oral amoxicillin (84.1%), with a cure rate of 82.2%. Children treated with cotrimoxazole (60% cure rate) and erythromycin (26.7% cure rate) had lower success rates. A higher proportion of girls (81.4%) were cured compared to boys (71.5%), and children who were fully vaccinated (79.6%) had a higher cure rate than those who were partially or not vaccinated (48.0%). Children of educated parents had higher cure rates than those of uneducated parents.

**Conclusions:**

Children with chest indrawing pneumonia were successfully treated with oral amoxicillin in an outpatient setting, improving access to treatment and reducing costs for both health care systems and families. This approach also helps reduce the risk of healthcare-associated infections. It is essential that health care providers in primary health facilities are trained to use IMCI protocols when managing children under five.

Pneumonia is one of the leading causes of morbidity and mortality in children under five years of age globally [1,2]. It accounted for 725 557 deaths in 2021, and while this presents a substantial reduction from the 1.59 million deaths in 2000 [3], the burden remains high, especially in low- and middle-income countries (LMICs) [4]. Zambia has a high burden of child pneumonia, with approximately 6000 children under five years of age dying of this condition every year. This comprises 15% of all deaths in this age group [5] and is a major contributor to the country’s high under-five mortality rate of 61 per 1000 live births [6]. Additionally, acute respiratory infections in Zambia account for 30–40% of children’s outpatient attendance and 20–30% of hospital admissions. Progress in reducing pneumonia-related child mortality in Zambia has been much slower than for other infectious diseases and may prevent the country from attaining the 2030 Sustainable Development Goal (SDG) target of 25 deaths per 1000 live births [7].

Effective management of childhood pneumonia is critical for reducing morbidity and mortality in children [8]. The World Health Organization (WHO) revised its pneumonia management guidelines in 2012 to specify that children aged 2–59 months with fast breathing and/or lower chest indrawing, without any danger signs, be treated on an outpatient basis with oral amoxicillin [9]. Lower chest indrawing is when a child's lower chest wall moves inward when they breathe in; it is a sign of respiratory distress and occurs when a child needs to exert more effort than normal to breathe in [9]. In 2014, the WHO revised the Integrated Management of Childhood Illness (IMCI) chart booklet accordingly. IMCI is a comprehensive approach to the management of common childhood illnesses (including pneumonia management) through prompt assessment, classification, appropriate treatment with antibiotics, and referral for severe cases with danger signs [10].

Despite the adoption of the revised pneumonia guidelines in Zambia and the additional revision of the IMCI chart booklet for the country’s context [11], limited information exists about its implementation in a public health setting the country. We aimed to evaluate outcomes two weeks after the initiation of treatment on an outpatient basis among children aged 2–59 months with chest indrawing pneumonia presenting at primary health care (PHC) facilities in Zambia.

## METHODS

### Study design

This was a prospective, observational cohort study conducted between October 2022 and April 2024 at various sites in Ethiopia, India, Pakistan, Nigeria, Uganda, and Zambia on children aged 2–59 months who presented at selected PHC facilities with chest indrawing pneumonia. Eligible participants were enrolled and managed by facility health care providers trained in IMCI and followed up on day 15 to record the treatment-related information and vital status, including conducting verbal autopsies in case of child death [12]. Zambia was selected due to its high prevalence of childhood pneumonia, its updated national policy to recommend oral amoxicillin at the outpatient level for treatment of chest indrawing pneumonia in children aged 2–59 months, and the availability of functional primary-level health facilities with staff trained in the updated WHO IMCI case management tool [12].

### Study setting

We selected eight PHC facilities from three districts (Lusaka, Chibombo, and Chongwe). Of these eight, four were rural health facilities – Chikobo, Mwachisopola, Chainda, and Chalimbana, with a catchment population of 10 682, 9652, 14 775, and 5616, respectively. Chaisa, Chazanga, Kanyama West, and Mtendere health facilities were located in urban areas with catchment populations of 90 953, 47 030, 84 700 and 94 759, respectively. These facilities were selected based on the high burden of childhood pneumonia and the presence of staff trained on the updated IMCI protocol. All participating facilities provided 24-hour outpatient services [12].

### Study population

Children aged 2–59 months presenting with cough and/or breathing difficulty and lower chest indrawing who lived in the study catchment area and whose parents/caregivers provided consent were eligible for enrolment. They were followed up on day 15 for study outcomes. We excluded children aged <2 months or >5 years; those showing any general danger signs (convulsions, inability to drink or breastfeed, vomiting everything, lethargy, unconsciousness), any other signs that would lead to severe classification such as stridor when calm, or any other severe classification such as severe malnutrition; those living in an area where the follow-up was not feasible; or those enrolled in another study at the time of the current study [12].

### Initial assessment

At the PHC facilities, the IMCI-trained health care providers assessed and classified children presenting with cough or difficulty breathing according to the IMCI chart booklet [11]. The health care providers classified children as having cough or cold (no pneumonia), pneumonia (fast breathing and/or chest indrawing), or severe pneumonia (any general danger sign, or stridor when calm). Fast breathing pneumonia was classified if infants 2–11 months had 50 breaths per minute or more, or if a child aged 12–59 months had 40 breaths per minute or more, while chest indrawing pneumonia was classified as the presence of chest indrawing, *i.e.* a child's lower chest wall moving inward when they breathed in. Healthcare providers assessed all children under five years of age and recorded their name, age, complete address, sex, place of residence, nutrition status (weight and height for all children, and mid-upper arm circumference (MUAC) of children aged six months or above), and illness classification, as well the caregivers’ education and telephone number. Treatment outcome and antibiotic administration. In parallel, research assistants (RAs) collected information on the vaccination status of all children (even those without pneumonia) and obtained informed written consent from parents/caregivers of children with pneumonia. Children whose parents gave consent were then enrolled in the study [12].

### Follow-up and outcome assessment

On day 15 after enrolment, the RAs (not involved in the classification and treatment of the enrolled children) contacted the parents or caregivers by phone to make an appointment for the follow-up visit, during which they recorded the child's survival status (primary outcome). They gathered information regarding the child’s treatment during the illness, including the name, route of administration, duration and frequency of antibiotic use, treatment adherence, and whether the child was treated on an outpatient basis or referred for hospitalisation.

The RAs also asked mothers/caregivers about the current condition of their child on day 15 (‘cured’, ‘same’, ‘worse’ and ‘not fully recovered but better compared to enrolment’). The window period for the day-15 follow-up was within a range of ±2 days. They also recorded this information on a pre-specified case report form [12].

### IMCI refresher training

Before implementing the study, two health care providers at each selected PHC facility underwent a one-day refresher training on IMCI in a classroom. The training focussed on assessing and classifying children aged 2–59 months presenting with cough and/or difficult breathing. To facilitate enrolment, all health workers were informed of the study’s objectives, design, methods, consent procedures, and ethical considerations regarding data collection.

### Study outcomes

The primary study outcome was the survival status of all enrolled children on day 15. Surviving children were further classified as ‘cured’, ‘same’, ‘worse’ and ‘not fully recovered but better compared to enrolment’ based on the mother/caretaker’s description on day 15. Antibiotic adherence was defined as children taking a full course of oral amoxicillin for a duration of five days or more.

### Sample size

Using the standard formula for a single proportion [13] with a 5% case fatality ratio (CFR) for chest indrawing, a 3% margin of error, and 95% confidence level, we calculated the sample size for the descriptive analysis to be 292 for this study. Assuming that 5% would be lost to follow-up, 310 children 2–59 months of age with chest indrawing pneumonia were to be enrolled and followed up [12].

### Data management and analysis

We used the Kobo electronic data collection tool to collect the study data, whereby we created a central data repository within a Kobo collect project data account through which the study facilities shared the data daily during the study period. We ran periodical data checks and corrections to minimise missing data. We did not perform imputation of missing data.

We analysed data using Stata, version 18.0 (StataCorp LLC, College Station, Texas, USA). We presented categorical variables such as sex, treatment received and vital outcome as frequencies and percentages, and continuous variables such as age as means and standard deviations or medians and interquartile ranges.

### Ethical considerations

We obtained ethical approval from the University of Zambia Biomedical Research Ethics Committee (UNZABREC ref: No. 2505-2022), the National Health Research Authority Zambia (NHRA Ref: NHRA000024/04/03/2022), and the WHO Ethical Review Committee. We also obtained permission to undertake the study from the relevant departments (Ministry of Health Headquarters, Provincial Health Office, District Health Office, and the participating PHC facility in charge). We translated all consent forms into local languages and obtained informed written consent from the parents/caregivers of all eligible children, while ensuring confidentiality and privacy for the whole study process.

## RESULTS

**Figure 1:**
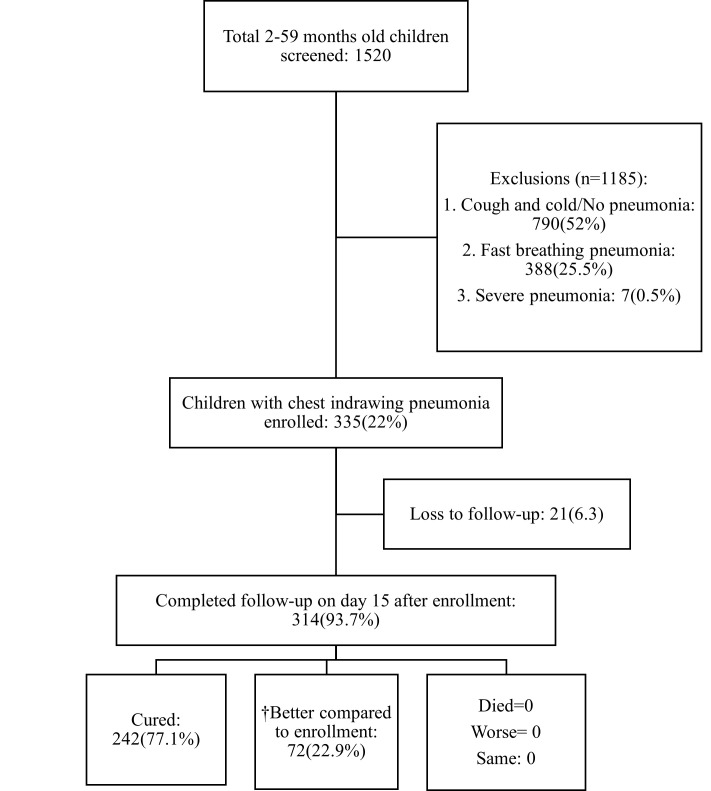
Flowchart of children screened, enrolled and analysed. *Fast breathing: children 2–11 months old with a respiratory rate of 50 breaths or more per minute; children 12–59 months old with a respiratory rate of 40 breaths or more per minute. †Better compared to enrollment – better than before, but not fully recovered.

We screened 1520 children aged 2–59 months, of whom 335 fulfilled the inclusion criteria and were enrolled in the study. We did not receive responses from the carers of 21 children. We followed up on 314 (93.7%) children on day 15 (**Figure 1**); all survived. Based on mothers’/caregivers’ statements, 242 (77.1%) were classified as ‘cured’ and 72 (22.9%) as ‘better compared to enrolment, but were not fully recovered’. None were categorised as ‘same’ or ‘worse’ compared to the time of enrolment.

### Sociodemographic and clinical characteristics

Of 335 children enrolled, 189 (56.4%) were female, 211 (63.0%) were aged 12–59 months, 79 (23.6%) had a height/length for age (HAZ) Z-score of<−3, 35 (10.5%) had a weight for height/length (WHZ) Z-score of<−3 (**Table 1**), and 289 (92.0%) had been fully vaccinated. Most mothers or female caregivers were educated; specifically, 165 (52.5%) had secondary or higher education and 130 (41.4%) had primary education. The situation was similar for fathers/male caregivers, whereby 224 (71.3%) had secondary education and 70 (22.3%) had primary education.

**Table 1:** Sociodemographic and clinical characteristics of enrolled children aged 2–59 months with chest indrawing pneumonia in Zambia (n = 335)*

Child age in months, MD (IQR)	15 (8.0–26.0)
**Child's age group in months**	
12–59	211 (63.0)
2–11	124 (37.0)
**Sex of child**	
Female	189 (56.4)
Male	146 (43.6)
**Respiratory rate/min (age in months), MD (IQR)**	
2–11	55 (50.5, 60.5)
**Respiratory rate/min (age in months), MD (IQR)**	
2–59	51 (44, 60)
**HAZ z-score**	
≥−2	220 (65.7)
<−2 to≥−3	36 (10.8)
<−3	79 (23.6)
**WHZ z-score**	
≥−2	284 (84.8)
<−2 to≥−3	16 (4.8)
<−3	35 (10.5)
**MUAC in cm†**	
≥12.5	262 (78.2)
11.5< to <12.5	16 (4.8)
≤11.5	3 (0.9)
<6 months old	54 (16.9)
**Rural health facilities**	217 (64.8)
Chikobo Rural Health Centre	84 (25.1)
Chalimbana Rural Health Centre	23 (6.9)
Mwachisopla Rural Health Centre	54 (16.1)
Chainda Rural Health Centre	56 (16.7)
**Urban health facilities**	118 (35.2)
Chaisa Urban Health Centre	16 (4.8)
Chazanga Urban Health Centre	40 (11.9)
Kanyama West Urban Health Centre	46 (13.7)
Mtendere Urban Health Centre	16 (4.8)
**Children followed up on day 15 (n = 314)**	
Child's vaccination status	
*Fully vaccinated*	289 (92.0)
*Partially/not vaccinated*	25 (8.0)
Source of vaccination information	
*Vaccination card*	250 (79.6)
*Verbal/reported*	64 (20.4)
Mother’s education level	
*Secondary and higher*	165 (52.5)
*Primary*	130 (41.4)
*No education*	19 (6.1)
Father’s education level	
*Secondary and higher*	224 (71.3)
*Primary*	70 (22.3)
*No education*	20 (6.4)

HAZ – height/length for age, IQR – interquartile range, MD – median, MUAC – mid upper arm circumference, WHZ – weight for height/length

*Values are n (%) unless specified otherwise.

†MUAC was not measured in children less than six months old.

Among 314 children who were followed on day 15, 264 (84.1%) had been treated with oral amoxicillin, 35 (11.2%) with oral cotrimoxazole, and 15 (4.8%) with oral erythromycin (**Table 2**). More than 97% (n/N = 257/264) of those taking oral amoxicillin did so for five days or more, over 91% (n/N = 31/35) taking oral cotrimoxazole did so for five days or more, while none completed five days of oral erythromycin.

**Table 2:** Adherence to oral antibiotic treatment (n = 314)*

Duration of treatment in days	Total (n = 314)	Age of child, 2–11 months (n = 122)	Age of child, 12–59 months (n = 192)
Oral amoxicillin	264 (84.07)†	102 (83.60)	162 (84.38)
*Less than 5*	7 (2.65)‡	1 (0.98)‡	6 (3.70)‡
*5*	218 (82.57)	90 (88.24)	128 (79.01)
*More than 5*	39 (14.77)	11 (10.78)	28 (17.28)
Oral cotrimoxazole	35 (11.15)†	15	20
*Less than 5*	4 (11.43)	2 (13.33)	2 (10.00)
*5*	23 (67.71)	9 (60.00)	14 (70.00)
*More than 5*	8 (22.86)	4 (26.67)	4(20.00)
Oral erythromycin	15 (4.78)†	5	10
*Less than 5*	15 (100.0)	5 (100.0)	10 (100.0)

*Presented as n (%).

†Denominator: n = 314.

‡Denominator: number of children who received that specific antibiotic.

### Comparison of children with chest indrawing pneumonia perceived to have been cured to those perceived as ‘better compared to enrolment’ (not fully recovered)

Children who were cured had a median age of 15.0 months, while those who were better compared to enrolment had a median age of 12.5 months (**Table 3**). More female children (n/N = 144/177, 81%) were cured than male children (n/N = 98/137, 71.5%). The children who had been fully vaccinated were more likely to be cured (n/N = 230/289, 79.6%) compared to those partially or not vaccinated (n/N = 12/25, 48%). Children were more likely to be perceived as cured if parents were educated (mothers: n/N = 231/295 (78.3%); fathers: n/N = 233/294 (79.3%)) than if they were uneducated (mothers: n/N = 11/19 (57.9%); fathers: n/N = 9/20 (45%)). The same pattern was seen among children who were better compared to enrolment but not ‘cured’ completely. Among children who took oral amoxicillin, 217/284 (82.2%) were cured compared to 21/35 (60%) treated with oral cotrimoxazole and 4/15 (26.7%) treated with oral erythromycin. More children who had been treated at rural health facilities (n/N = 174/208, 83.7%) were perceived as cured, compared to those treated in urban centres (n/N = 68/106, 64.2%).

**Table 3:** Comparison of children with chest indrawing pneumonia perceived to have been cured to those perceived as ‘better compared to enrolment’ (not fully recovered)*

Variable	Perceived treatment outcome		
**Total (n = 314)**	**Cured (n = 242)**	**Better compared to enrolment, but not fully recovered (n = 72)**
**Child’s age in months, MD (IQR)**	14	15.0 (8.0, 27.0)	12.5 (6.0, 22.5)
**Child's age group in months**			
12–59	192	152 (79.2)	40 (20.8)
2–11	122	90 (73.8)	32 (26.2)
**Sex of child**			
Female	177	144 (81.4)	33 (18.6)
Male	137	98 (71.5)	39 (28.5)
**Vaccination status**			
Fully vaccinated	289	230 (79.6)	59 (20.4)
Partially/not vaccinated	25	12 (48.0)	13 (52.0)
**HAZ z-score**			
≥−2	212	168 (79.2)	44 (20.8)
<−2 to ≥−3	33	23 (69.7)	10 (30.3)
<−3	69	51 (77.1)	18 (22.9)
**WHZ z-score**			
≥−2	264	206 (78.0)	58 (22.0)
<−2 to ≥−3	16	10 (62.5)	6 (37.5)
<−3	34	26 (76.5)	8 (23.5)
**MUAC in cm (n = 261)**†			
≥12.5	249	197 (79.1)	52 (20.9)
11.5≤ to <12.5	10	7 (70.0)	3 (30.0)
<11.5	2	0 (0.0)	2 (100.0)
**Given oral antibiotic**			
Amoxicillin	264	217 (82.2)	47 (17.8)
Cotrimoxazole	35	21 (60.0)	14 (40.0)
Erythromycin	15	4 (26.7)	11 (73.3)
**Adherence to oral antibotics**			
Adherence to oral amoxicillin			
*Adherent*‡	257	212 (82.5)	45 (17.5)
*Non-adherent*	7	5 (46.2)	2 (53.9)
**Adherence to other antibiotics**			
*Adherent*‡	31	18 (58.1)	13 (41.9)
*Non-adherent*	19	7 (36.8)	12 (63.2)
**Mother’s education level**			
Secondary and higher	165	142 (86.1)	23 (13.9)
Primary	130	89 (68.5)	41 (31.5)
No education	19	11 (57.9)	8 (42.1)
**Father’s education level**			
Secondary and higher	224	190 (84.8)	34 (15.2)
Primary	70	43 (61.4)	27 (38.6)
No education	20	9 (45.0)	11 (55.0)
**Health facilities**			
Rural	208	174 (83.7)	34 (16.3)
Urban	106	68 (64.2)	38 (35.8)

HAZ – height/length for age, IQR – interquartile range, MD – median, MUAC – mid upper arm circumference, WHZ – weight for height/length

*Presented as n (%) unless specified otherwise.

†MUAC was not measured in children less than six months old.

‡IMCI recommends oral amoxicillin for five days. For this study, children who received antibiotics for five days or more were considered adherent.

## DISCUSSION

All children in our study survived until day 15, and none worsened after recruitment. According to their mothers, some had still not fully recovered, but had improved from their initial presentation. We found that most children were treated with oral amoxicillin and that they were more likely to be cured than those treated with other oral antibiotics. We also found that a greater proportion of children with chest indrawing were cured when they were fully vaccinated for their age, *i.e.* according to their vaccination schedule, when their parents were educated, when they were female, and when they received treatment at rural health facilities. Only 6.3% of children were lost to follow-up.

No deaths were reported in our study. This could be related to early recognition of signs of pneumonia, appropriate care-seeking, and provision of correct treatment by trained health care providers, and high compliance with the prescribed antibiotic treatment. It has been demonstrated that standard case management of common childhood illnesses reduces child mortality [14–16]. Our results are similar to an observational study carried out at the outpatient level in Papua New Guinea [17], which reported no deaths among 117 children with chest indrawing pneumonia who were managed with oral amoxicillin at home. Another outpatient-based multicentre observational study in four countries [18] reported no deaths among 873 children with chest indrawing pneumonia treated with oral amoxicillin at home. A community-based observational study from Kenya where community-level health workers treated with oral amoxicillin and followed up 1799 children with chest indrawing pneumonia; reported only 5 (0.3%) deaths at 14 days [19]. In two community-based randomised controlled trials (RCTs) in Pakistan [20,21] and an RCT in four African and Asian countries [22] where community-level health workers treated chest indrawing pneumonia at home with oral amoxicillin, the case fatality ratio (CFR) was negligible (<1%). Similarly, several large hospital-based multicentre RCTs in India [23], in Kenya [24], in Pakistan [25] and in eight countries [26] reported <1% CFR, where a large number of children with chest indrawing were treated with oral amoxicillin at home.

Concerns have been raised that the revised WHO guidelines recommending treatment of chest indrawing pneumonia in children with oral amoxicillin at home were only based on data from RCTs and thus did not account for the real-life settings in LMICs, which could result in higher pneumonia mortality [27,28]. The concern about low CFR from RCTs is understandable because recruited patients are selected and managed carefully in a controlled setting. We conclude that real-life observational studies that report similar results in hospital outpatient settings [17,18], communities [19], and our study in PHC facilities can alleviate such concerns. We believe that the WHO-revised guidelines [9] are appropriate and safe to implement in low-resource settings, and will contribute to both an increase in access to treatment and to a reduction in pneumonia mortality in Zambia and elsewhere.

We found that the health providers' adherence to the IMCI recommendation to treat chest indrawing pneumonia with oral amoxicillin was high compared to other studies [29-32]. However, children with severe acute malnutrition were not referred as per the IMCI chart booklet recommendation. This non-adherence observed in studies from other locales [29–32] is not always due to a lack of knowledge; it may also be due to a lack of motivation to adhere to the IMCI tool, or a lack of capacity to concentrate fully on each case, which has sometimes been attributed to poor remuneration [33].

In additional comparisons, our loss to follow-up (6.3%) was relatively similar to the 5.6% reported in an observational community-based study in Kenya [19], as well as to the 4.1% from a multicountry observational outpatient-based study [18]. It is lower than the 12.8% reported in an observational outpatient-based study in Papua New Guinea [17] and higher than the 2.5% [22] and 2.4% [20] reported in community-based RCTs. In our study, the prevalence of pneumonia was higher in rural compared to urban settings, which is similar to the data reported from Uganda and Ethiopia [34,35]. While this difference could be due to the lower socioeconomic status and lower education levels in rural areas, others have reported that pneumonia prevalence is more common in urban areas, primarily due to overcrowding and having an urban place of residence [36].

Managing children with chest indrawing pneumonia on an outpatient basis with oral antibiotics treatment at home is likely to reduce costs for both the health system and families. A systematic analysis of the costs of managing pneumonia [37] reported that the cost of pneumonia treatment was USD 4.3 in the community, USD 51.7 at an outpatient facility, and USD 242.7 at different levels of hospitals. A study from Zambia reported the cost of pneumonia to be USD 48 per hospital visit and USD 215 per bed day in hospital [38], which is extremely high. Zhang and colleagues [39] carried out a cost-effective analysis that compared the 2005 WHO pneumonia management guidelines [40] with the 2012 revised WHO guidelines [9] in 74 LMICs. They found that, besides being cost-effective, the revised guidelines could generate substantial cost savings for low-resource countries. For Zambia, the Zhang study showed a reduction in direct medical costs for the management of pneumonia following the 2012 WHO guidelines compared to the 2005 guidelines. The lower costs are mainly due to the reduction in hospital care, as most pneumonia cases with lower chest indrawing would be treated on an outpatient basis instead of in hospital. Moreover, the direct cost for the families for treatment of pneumonia was reported by Madsen and colleagues [41] to be USD 41.35 at a secondary-level hospital and USD 134.62 at a tertiary-level hospital [41]. Another study reported that the direct out-of-pocket expenditure for a hospitalised child with respiratory infection was 34% of the annual per capita income [42]. Hussain and colleagues [43] reported that medicines constituted the highest proportion of household expenditures, followed by meals, hospitalisation, and transportation. Thus, the revised 2012 WHO guidelines are not only cost-effective for the health system, but also beneficial for families through reduced transport costs, meal costs, hospitalisation, loss of wages, and other related costs [39,43,44].

In our study, the IMCI-trained health workers used standard case management practices to identify and manage pneumonia and other common childhood illnesses [11]. It has been demonstrated that standard case management for common illnesses rationalises and reduces antibiotic use [14,45]. This has significant implications for the national programmes in Zambia and elsewhere. We know that increased use of antibiotics can lead to an increase in antimicrobial resistance (AMR), which is a huge global public health problem. It is estimated that 7.7 million deaths globally are attributed to bacterial infections, of which nearly five million are associated with drug-resistant pathogens [46,47]. In sub-Saharan Africa, antibiotics are easily available in many communities through shops and pharmaceutical stores; they are not only used widely in homes, but also in commercial animal husbandry, leading to increasing antimicrobial resistance [48]. It has been estimated that the all-age death rate attributable to AMR is highest in western sub-Saharan Africa, at 27.3 deaths per 100 000 [47]. WHO has launched the WHO Access, Watch, Reserve (AWaRe) framework [49], which gives specific guidance on the empirical use of antibiotics according to the WHO essential medicines list [50] focussed on the risk of AMR development associated with the use of different antibiotics. Oral amoxicillin, included in the ‘Access’ list of medicines, is recommended for the treatment of pneumonia in children [49]. Thus, its appropriate use to treat pneumonia on an ambulatory basis must be promoted to combat this global public health problem, particularly in sub-Saharan Africa.

All the patients in our study were treated at home with oral antibiotics. This contrasts the 2005 WHO guideline that recommended that chest indrawing pneumonia be referred for hospitalisation and treatment with injectable antibiotics [40]. Healthcare-associated infections result in substantial morbidity and mortality in hospitals, and were reported to be higher in LMICs compared to high-income countries [51,52]. One study reported the frequency of health care-associated infections to be highest in Latin America and East and South Asia [53], where an antibiotic was most commonly prescribed for pneumonia (19.2%). A recent analysis of point prevalence surveys from 99 countries reported the global number of health care-associated infections per year to be 136 million, with the highest burden observed in China, Pakistan, and India [54]. This is another important reason to promote ambulatory treatment of most cases of pneumonia with oral antibiotics in a programme setting.

Findings from our study demonstrate the benefits of the IMCI approach to managing cases in outpatient situations in health facilities. The treatment of chest indrawing pneumonia in an outpatient setting will reduce the government expenditure on referrals and inpatient care, as well as the costs for the families, and will also help lower the AMR pressure and the prevalence of health care-associated infections. The findings are crucial to informing practice and policy, particularly in low-resource settings such as Zambia. Specifically, they are important in the review and updating of existing policy documents and guidelines such as the National Formulary for Drug Use, as well as for enhancing capacity building and mentorship of health care providers. They will also provide further evidence for the continued procurement of oral essential antibiotics such as amoxicillin.

This study has several strengths. First, consideration of multiple rural and urban settings allows for the generalisation of the results to similar resource-limited settings. However, some limitations should also be noted. Mothers were interviewed two weeks after initial recruitment, so the time lag may have introduced a recall bias, as well as social desirability bias. Outcomes were based on mothers’ reports and mothers’ perceptions; this might have played a role in the final categorisation, especially among those children who were declared not fully recovered. We did not establish the aetiology of the pneumonia patients (bacterial, viral or mixed infections). Further, we did not test for associations, which limits the ability to make definitive claims in this sense; we recommend that future studies take this into account. Finally, including more information on comorbidities such as malaria, HIV/AIDs and diarrhoea would have been useful, as it could have explained why some children were not fully recovered by the follow-up visit.

## CONCLUSIONS

Children with chest indrawing pneumonia were successfully treated with oral amoxicillin given by mothers at home. No adverse outcomes were observed. This suggests the need for continued efforts in promoting this approach, including by training all health care providers in PHC facilities. Targeted interventions focussing on unvaccinated children are crucial in efforts to leave no one behind and to reach the SDGs, particularly in resource-constrained settings. Last, but not the least, there is a need to ensure that IMCI trainings and refresher courses are enhanced for continuous improvement of outpatient chest indrawing pneumonia management among health care providers.
